# Isolation and Preliminary In Vitro Characterization of a Honey-Derived *Bacillus licheniformis* Isolate

**DOI:** 10.3390/antibiotics15090832

**Published:** 2026-08-27

**Authors:** Mehtap Usta, Kübra Zengin, Yakup Kara, Remziye Nalçacıoğlu, Zihni Demirbağ, Oktay Yıldız, Sevgi Kolaylı

**Affiliations:** 1 Tonya Vocational School, Trabzon University, 61500 Trabzon, Türkiye; mehtapyakupoglu@trabzon.edu.tr; 2 Department of Biology, Science Faculty, Karadeniz Technical University, 61080 Trabzon, Türkiye; kubrazngn97@gmail.com (K.Z.); remziye@ktu.edu.tr (R.N.); zihni@ktu.edu.tr (Z.D.); 3 Department of Chemistry, Science Faculty, Karadeniz Technical University, 61080 Trabzon, Türkiye; yaakupkara@gmail.com; 4 Faculty of Pharmacy, Karadeniz Technical University, 61080 Trabzon, Türkiye; oktayyildiz29@hotmail.com; 5 Okta R & D Eng Services Industry Trade Limited Company, Trabzon Teknocity, 61081 Trabzon, Türkiye; 6 Gümüşhane University, Rectorate, 29100 Gümüşhane, Türkiye; 7 Department of Biochemistry, Faculty of Medicine, Nakhchivan State University, AZ7012 Nakhchivan, Azerbaijan

**Keywords:** honey, *Bacillus licheniformis*, probiotic bacteria

## Abstract

**Background/Objectives:** Despite its hostile physicochemical environment characterized by high osmotic pressure, acidic pH, and low water activity, honey serves as a unique ecological niche for resilient microorganisms. This study aimed to recover bacterial isolates from honeys of different botanical origins and to characterize the probiotic-associated properties of a honey-derived *Bacillus licheniformis* isolate. **Methods:** A total of 20 bacterial isolates were recovered from four honey samples, including two blossoms and two honeydew honeys. 16S rRNA gene sequence analysis showed that the recovered isolates had taxonomic assignments consistent with *Bacillus licheniformis*. Because 16S rRNA sequencing does not provide sufficient resolution to establish strain-level diversity or clonality, one isolate, designated Blhoney, was selected for subsequent molecular and functional characterization. **Results:** In vitro assays revealed that Blhoney exhibits robust tolerance to simulated gastrointestinal stress, including extreme acidity (pH 1–3), bile salts (0.3–1%), pepsin, and pancreatin, maintaining viability for up to 24 h. Phenotypic antimicrobial testing demonstrated measurable inhibition zones for erythromycin, kanamycin, chloramphenicol, ampicillin, tetracycline, gentamicin, streptomycin, and spectinomycin, whereas no measurable inhibition zones were observed for penicillin G, rifampicin, ciprofloxacin, or ceftazidime. However, MIC determination and standardized breakpoint interpretation were not performed; therefore, these findings were interpreted solely as preliminary phenotypic inhibition-zone observations. Although the isolate did not display direct antagonistic activity against the tested bacterial pathogens under experimental conditions, its persistence under the tested gastrointestinal stress conditions supports further investigation of its probiotic-associated characteristics. **Conclusions:** Overall, these findings indicate that the honey-derived Blhoney isolate exhibits preliminary probiotic-associated characteristics under the tested in vitro conditions. However, further strain-level genomic and safety characterization is required before its potential probiotic application can be established.

## 1. Introduction

Honey is a natural product produced by honey bees (*Apis mellifera*) through the transformation of blossom nectar or different secretions. Due to its complex composition, including sugars, organic acids, enzymes, phenolic compounds, and other bioactive molecules, honey is recognized for its food and therapeutic properties [1,2]. The physicochemical properties of honey, particularly its low water activity, acidic pH, and high osmotic pressure, create an environment that inhibits the growth of many microorganisms and allows only specific microbial communities to survive [3,4,5]. As a result, honey occupies a unique ecological niche capable of harboring microorganisms that can withstand extreme environmental conditions.

Although honey possesses strong antimicrobial properties, several studies have demonstrated that it still contains diverse microbial populations originating from nectar, pollen, environmental sources, and the digestive tract of honeybees [6,7]. Among these microorganisms, lactic acid bacteria (LAB) have attracted particular attention due to their functional roles in the honeybee microbiome and their potential applications in biotechnology and human health. LAB associated with honeybees and bee products have been shown to produce antimicrobial metabolites such as organic acids, bacteriocins, and hydrogen peroxide, which contribute to the inhibition of pathogens and the maintenance of hive health [7,8]. In addition, these bacteria exhibit remarkable tolerance to acidic and osmotic stress conditions, suggesting that bee-related environments may represent promising reservoirs of robust probiotic microorganisms.

Probiotics are defined as live microorganisms that confer health benefits on the host when administered in adequate amounts [9]. While most commercially available probiotic strains have traditionally been isolated from fermented dairy products or the gastrointestinal tracts of humans and animals, increasing attention has recently been directed toward unconventional ecological niches for the discovery of novel probiotic candidates. Bee products such as honey, pollen, propolis, and royal jelly represent particularly promising environments due to the presence of microorganisms adapted to extreme physicochemical conditions and capable of producing antimicrobial compounds [10,11]. Consequently, the exploration of bee-derived microbial communities may provide new opportunities for identifying functional probiotic bacteria with potential applications in food, health, and biotechnology.

Honeys produced by the honeybee (*Apis mellifera*) are primarily classified into two distinct categories based on their botanical origin: blossom honey and honeydew honey. While honeys derived from floral nectars are defined as blossom honeys, those produced from the excretions of plant-sucking insects or secretions of living plant parts are termed honeydew honeys. One of the most discriminative physicochemical parameters used to differentiate these two types is specific optical rotation. Characteristically, blossom honeys exhibit levorotatory (negative) optical rotation, whereas honeydew honeys present dextrorotatory (positive) values [12,13].

Among the various types of honey, honeydew honey represents a distinct category produced not from floral nectar but from the sugary excretions of plant-sucking insects such as aphids and scale insects that feed on plant phloem sap. These insects secrete nectar droplets that are collected by honeybees and subsequently converted into honeydew within the hive. Due to their distinct origins, honeydew honey differs significantly from floral honey in terms of sugar composition. It typically exhibits a darker color, higher mineral content, higher electrical conductivity, and increased levels of phenolic compounds and oligosaccharides [11,13]. These physicochemical differences may influence the structure and diversity of microbial communities associated with nectar honey [14].

The microbial ecology of honeydew is shaped by complex ecological interactions between insects, microorganisms, and honeybees that feed on plant sap. Insect species feeding on plant phloem host various microbial symbionts that influence the biochemical composition of honeydew secretions [15]. When bees collect these secretions, microorganisms from plant surfaces, insect microbiota, and the hive environment can enter the honey matrix, potentially increasing microbial diversity and functional complexity [16]. Consequently, honeydew may constitute a unique ecological niche capable of harboring previously undiscovered microbial communities.

Despite the increasing number of studies investigating honey microbiota and bee-associated microorganisms, the majority of research has primarily focused on blossom honeys and the gut microbiota of honeybees. Consequently, the microbial composition and functional potential of honeydew honey remain relatively underexplored [17]. Existing studies have mainly addressed the physicochemical characteristics and antioxidant properties of honeydew honey, whereas investigations on its microbiological diversity and potential probiotic bacteria are still limited [18]. Considering the distinctive ecological origin and chemical composition of honeydew honey, it is plausible that this honey type may host unique microbial communities with significant probiotic potential that have not yet been thoroughly characterized.

Furthermore, recent studies have highlighted the growing interest in identifying microorganisms from natural environments that possess probiotic traits and biotechnological potential [19]. However, research focusing specifically on the isolation and functional characterization of probiotic bacteria from honeydew honeys is still scarce, indicating an important knowledge gap in the current literature. Within this framework, the present study aimed to isolate bacteria from blossom and honeydew honeys of different botanical origins, identify the recovered isolates, and perform detailed molecular and functional characterization of a one selected isolate. Particular emphasis was placed on gastrointestinal stress tolerance, antibiotic susceptibility, and antimicrobial activity as relevant characteristics for evaluating its potential probiotic properties. By exploring the unique microbial niche of honeydew honey, this research aims to identify resilient bacterial isolates exhibiting preliminary probiotic-associated characteristics, thereby expanding our current understanding of the microbiological and biotechnological potential of bee-derived products.

Accordingly, honey should be regarded not as a direct probiotic food but as a natural ecological source for the discovery of bacterial strains with potential probiotic properties that may subsequently be developed through controlled cultivation and appropriate safety assessment.

## 2. Results and Discussions

### 2.1. Physicochemical Properties

The botanical origins of the four honey samples used in this study were determined by microscopic pollen analysis. Physicochemical characterization was performed primarily to verify the botanical origin and quality attributes of the honey samples, thereby providing ecological context for the recovered bacterial isolates. The proportions of major, secondary, and minor pollen detected in the samples are presented in Table 1. Among the four samples, two were classified as blossom honey and two as honeydew honey were obtained from Türkiye in 2025 harvest seasons. Microscopic pollen analysis is one of the most widely used techniques for determining the botanical origin of honey. In the present study, pollen analysis based on pollen morphology under light microscopy revealed that two of the analyzed honey samples were rich in pollen content, and one of these was identified as monofloral rhododendron honey [20].

The H1 sample, designated as oak honey, exhibited a relatively low pollen density, with *Quercus* spp. pollen primarily classified as minor pollen. The H2 sample, collected from the Şalpazarı region in the Black Sea area, where Rosa canina (forest rose) flora predominates, was identified as a monofloral forest rose honey. The H3 sample, produced in Balıkesir, was classified as a multifloral blossom honey. The fourth sample, obtained from İkizdere in Rize during September, was identified as a honeydew honey, characterized by a very low pollen density and the absence of a dominant pollen type. Rhododendron honey, also known as ‘mad honey’, contains grayanotoxins, which are responsible for its toxic properties; therefore, its consumption is generally reported to be limited or carefully controlled [21,22]. From a botanical perspective, honey is typically grouped into two main types: nectar-based (blossom) honey and honeydew-derived honey.

Blossom honeys are produced when honeybees collect nectar from flowering plants and convert it into honey through enzymatic processes and dehydration within the hive. Examples of blossom honeys include chestnut, acacia, thyme, lavender, and rhododendron honeys. In contrast, honeydew honeys are produced from sugary secretions obtained from non-floral plant sources [23]. These secretions may originate either from excretions of plant-sucking insects (such as aphids) that feed on plant sap, or from sweet exudates released directly by plants. Pine honey and some forest honeys are typical examples of honeydew honeys. Oak honey is classified as a honeydew honey and is produced from sugary exudates secreted by oak trees (*Quercus* spp.), which are released as a physiological response to environmental factors such as day–night temperature fluctuations. These honeys are typically characterized by low pollen content and positive specific optical rotation values [24]. Another important parameter for distinguishing blossom and honeydew honeys is electrical conductivity. Generally, blossom honeys exhibit electrical conductivity values below 0.80 mS/cm, with the exception of chestnut honey, which may present higher values [23,24].

Moisture content among the honey samples was found to range from 17.50% to 18%, whereas electrical conductivity values ranged from 0.34 to 1.24 mS/cm. Specific optical rotation values were found to be negative in blossom honey samples and positive in honeydew honey samples. The proline concentrations of the honey samples were found to vary between 388 and 746 mg/kg. Total phenolic content, as an indicator of bioactive compounds, varied between 27.52 and 72.24 mg GAE/100 g honey. Proline content is considered an important quality marker for differentiating genuine honey from adulterated honey and is included among the quality criteria specified in the honey codex [25]. The relatively high proline levels detected in all analyzed honey samples indicate that these honeys comply with the quality requirements specified in the honey codex. Polyphenols constitute the major group of secondary metabolites responsible for the antioxidant properties of honey. In the present study, oak honey exhibited the highest total phenolic content (TPC) among the analyzed samples (Table 2). According to previous studies, there is a significant correlation between honey color and its phenolic content; darker honeys, such as oak honey, are generally characterized by higher levels of phenolic compounds [24]. Another notable characteristic of honeydew honeys is their tendency to crystallize more slowly compared to blossom honeys. This delayed crystallization contributes to the natural preservation and stability of these honeys.

### 2.2. Identification and Characterization of Bacterial Isolates Recovered from Honey

Microbiological examination of the four honey samples following cultivation on MRS agar resulted in the recovery of a total of 20 bacterial isolates, comprising 4 isolates from H1, 5 from H2, 6 from H3, and 5 from H4. The Blhoney isolate selected for subsequent molecular and functional characterization originated from H1, the oak honey sample collected from Çanakkale, Türkiye. Based on 16S rRNA gene sequence analysis, the recovered isolates showed taxonomic assignments consistent with *B. licheniformis*. The recovery of *B. licheniformis* despite the use of MRS enrichment medium highlights that this medium is not exclusively selective for lactic acid bacteria and may also support the growth of acid-producing spore-forming bacteria. However, because 16S rRNA sequencing does not provide sufficient resolution for strain-level discrimination within the *Bacillus subtilis/licheniformis* group, these findings do not establish whether the recovered isolates represent clonal replicates or genetically distinct strains. Therefore, one isolate, designated Blhoney, was selected for subsequent molecular and functional characterization. All subsequent functional findings reported in this study refer specifically to Blhoney and should not be generalized to the remaining recovered isolates.

**Figure 1:**
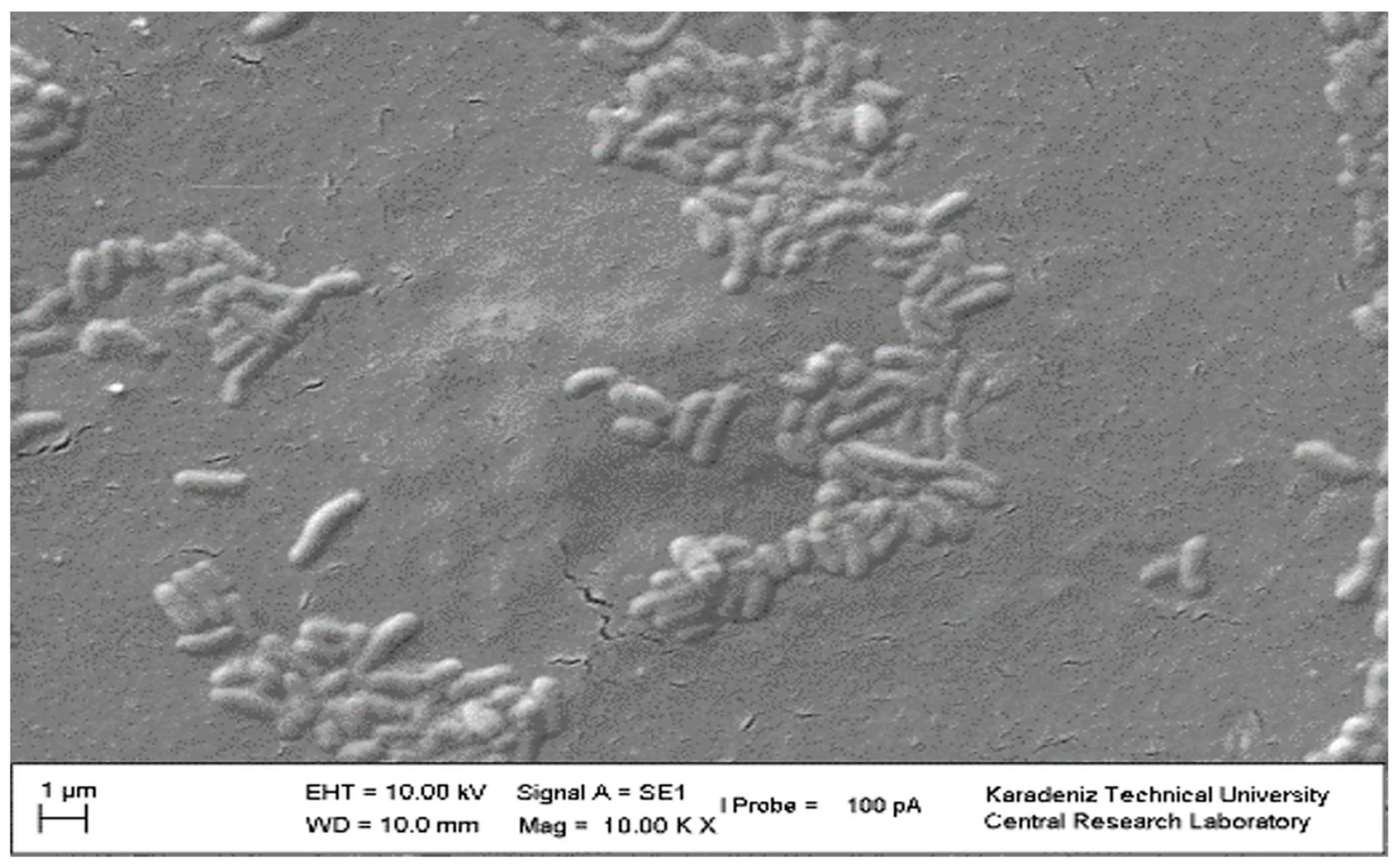
Morphological visualization of the Blhoney isolate obtained by scanning electron microscopy (SEM).

16S rRNA gene sequence analysis showed that Blhoney had its closest taxonomic assignment to *Bacillus licheniformis*, and the corresponding sequence was deposited in the GenBank database under accession number PZ120893. In addition, morphological observations obtained through scanning electron microscopy (SEM) were used to complement and support the molecular findings (Figure 1). The obtained 16S rRNA gene sequence (1540 bp) showed 98.90% identity with *Bacillus licheniformis* strain NR_074923.1 (GenBank accession no. OP001730).

Honey and honey-derived environments represent unique ecological niches that harbor diverse microbial communities capable of surviving under extreme environmental conditions such as high osmotic pressure, low water activity, and acidic medium. Recent culture-independent investigations further support this ecological perspective. Using a combined 16S rRNA bacterial and ITS2 fungal amplicon sequencing approach, the *Pathogens* study [26] demonstrated that honey harbors complex microbial communities whose composition is strongly influenced by botanical origin, environmental conditions, and beekeeping practices. The authors showed that both bacterial and fungal communities vary considerably among honey samples, supporting the concept that honey represents a dynamic microbial ecosystem rather than a sterile food matrix. Although the present study employed a culture-dependent approach focused on the isolation and characterization of cultivable bacteria, our findings are consistent with these observations by demonstrating that honey can serve as a natural reservoir of resilient microorganisms, including *Bacillus licheniformis*. Together, these studies reinforce the view that honey constitutes a valuable ecological niche for the discovery of microorganisms with potential functional and biotechnological relevance. Although honey has well-known antimicrobial properties, several studies have demonstrated that it can contain beneficial microorganisms, including lactic acid bacteria and spore-forming bacteria associated with honeybees and hive environments [3,7,10]. In the present study, bacterial isolates were successfully obtained from honeydew honey samples collected from different botanical origins, indicating that honeydew honey can serve as a reservoir of microorganisms with potential probiotic properties.

The isolation of *Bacillus licheniformis* from honey is consistent with previous reports demonstrating that honey and other bee-derived products represent ecological reservoirs of spore-forming bacteria capable of surviving under harsh environmental conditions. Although honey possesses strong antimicrobial activity due to its low water activity, acidic pH, and high osmotic pressure, several studies have shown that resilient *Bacillus* species can persist within this environment and contribute to its microbial diversity [17,18]. Similarly, bee-associated environments, including the honeybee gut, pollen, propolis, and royal jelly, have increasingly been recognized as valuable sources of microorganisms with potential probiotic and biotechnological applications [19,27].

Among the genus *Bacillus*, *B. licheniformis* has attracted considerable attention because of its spore-forming ability, environmental resilience, and capacity to survive conditions encountered during gastrointestinal transit. Previous studies have reported that *B. licheniformis* strains exhibit high tolerance to acidic environments and bile salts, characteristics that are considered essential prerequisites for probiotic functionality [28,29]. The high tolerance observed for the Blhoney isolate under the simulated gastrointestinal conditions employed in the present study is therefore consistent with the intrinsic physiological characteristics previously described for this species. The remarkable stress resistance of *Bacillus* species is largely attributed to their ability to produce highly resistant endospores together with multiple adaptive responses that enable survival under adverse environmental conditions [30,31].

Microbiological isolation yielded twenty bacterial isolates from the honey samples, from which one isolate, designated Blhoney, was selected for subsequent characterization. Genetic identification based on 16S rRNA sequencing confirmed its classification as *Bacillus licheniformis*. This finding is consistent with previous studies reporting that *Bacillus* species are commonly isolated from honey, bee products, and hive environments [6,18]. Spore-forming bacteria such as *Bacillus* spp. are known to survive in honey due to their ability to tolerate harsh environmental conditions, including osmotic stress and nutrient limitation [5]. In addition, several studies have highlighted that *B. licheniformis* possesses probiotic potential and has been investigated for its applications in food biotechnology and functional foods [30,31,32]. Furthermore, *B. licheniformis* has been reported to improve intestinal microbiota balance, immune responses, and growth performance in different animal models, supporting its potential as a probiotic microorganism [32,33,34]. Importantly, probiotic-associated properties of *B. licheniformis* are strain-dependent. Therefore, previously reported effects of other *B. licheniformis* strains provide biological context for the phenotype observed in the present study but cannot be assumed to apply to Blhoney without strain-specific experimental validation.

The probiotic potential of microorganisms largely depends on their persistence under the tested gastrointestinal stress conditions. In this study, the isolate demonstrated strong tolerance to acidic environments (pH 1–3), bile salts (0.3–1%), and digestive enzymes such as pepsin and pancreatin. These findings suggest that the isolate has the potential to survive during passage through the gastrointestinal tract. The uniformly high persistence observed under the simulated gastrointestinal conditions should be interpreted cautiously within the context of the experimental design employed in this study. *Bacillus licheniformis* is a spore-forming species, and previous studies have reported considerable tolerance of *B. licheniformis* strains to acidic and other environmental stresses. These characteristics provide biological context for the persistence observed for Blhoney under the applied in vitro conditions; however, they cannot be assumed to fully explain the phenotype of this isolate without strain-specific validation. Moreover, the present findings should not be interpreted as demonstrating survival during actual gastrointestinal passage in a host, but rather as evidence of high persistence under the specific simulated gastrointestinal conditions tested. Similar observations have been reported in previous studies where probiotic candidates isolated from honey and bee environments exhibited high tolerance to low pH and bile salts [35,36,37,38]. Such tolerance is considered one of the most important criteria for the selection of probiotic microorganisms because it determines their ability to reach the intestinal tract in viable form [34]. Furthermore, the high survival rates observed in this study may be related to the physiological adaptability of *Bacillus* species, which are known to form resistant endospores capable of surviving adverse environmental conditions [28].

Interest in *B. licheniformis* has also increased because several strains have demonstrated beneficial effects in animal models, including modulation of gut microbial communities, enhancement of intestinal barrier function, and stimulation of immune responses [32,33,34]. Nevertheless, these beneficial properties are recognized as highly strain-dependent, and not all *B. licheniformis* isolates possess equivalent probiotic characteristics or safety profiles. Consequently, each newly recovered isolate requires independent phenotypic and genomic evaluation before being considered for probiotic applications [27,29,39,40,41,42,43].

The Blhoney isolate exhibited high survival under the simulated gastrointestinal stress conditions, although modest variations were observed according to the stress condition and exposure duration (Table 3). At pH 1.0, survival decreased from 98.37 ± 0.31% after 1 h to 97.53 ± 0.31% after 24 h. After 24 h, survival at pH 2.0 and pH 3.0 was 97.30 ± 0.30% and 99.05 ± 0.49%, respectively. Similarly, after 24 h of exposure to 0.3%, 0.5%, and 1.0% bile salts, survival was 98.17 ± 0.15%, 98.37 ± 0.55%, and 98.10 ± 1.00%, respectively. The corresponding 24-h survival values following exposure to pepsin and pancreatin were 98.47 ± 0.55% and 98.50 ± 0.71%, respectively.

The isolate exhibited high survival following exposure to the tested acidic, bile salt, and enzymatic stress conditions, with modest variations according to stress condition and exposure duration (Table 3). These findings should be interpreted cautiously because the experimental design did not distinguish between vegetative cells and endospores.

Importantly, the experimental design did not distinguish vegetative cells from endospores. Therefore, the persistence observed under severe stress conditions, particularly prolonged exposure to highly acidic conditions, may partly or substantially reflect the survival of resistant endospores rather than continuous viability of vegetative cells. Since sporulation and subsequent germination were not quantified, the present findings cannot establish the proportion of metabolically active vegetative cells surviving gastrointestinal stress. Future studies should incorporate specific approaches for differentiating vegetative cells from endospores, including heat-treatment-based spore enumeration and germination assays, to determine the respective contributions of these physiological states to gastrointestinal stress tolerance.

In this study, one of the primary aims of comparatively analyzing samples with different botanical origins, namely honeydew and floral honeys, was to investigate whether the probiotic properties of honey are independent of its botanical source. Based on the obtained findings, bacterial isolates with 16S rRNA-based taxonomic assignments consistent with *B. licheniformis* were recovered from honey samples of different botanical origins. However, because only Blhoney underwent detailed functional characterization, the present study does not allow conclusions regarding the distribution of probiotic-associated characteristics among the different honey types or isolates.

### 2.3. Determination of Antibiotic Susceptibility and Antimicrobial Activity

**Figure 2:**
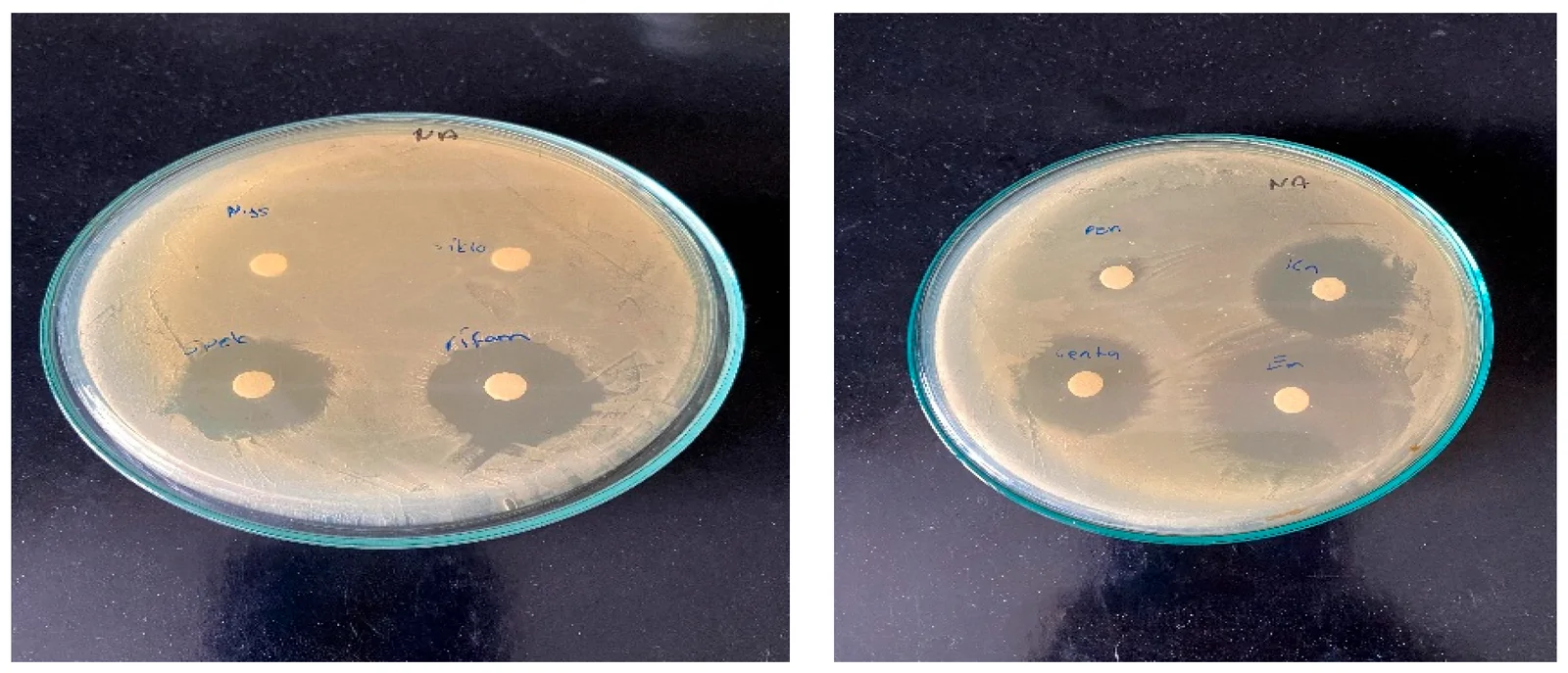
Antibiotic susceptibility test results of Blhoney.

Phenotypic testing of the Blhoney isolate demonstrated measurable inhibition zones for erythromycin, kanamycin, chloramphenicol, ampicillin, tetracycline, gentamicin, streptomycin, and spectinomycin under the applied experimental conditions, whereas no measurable inhibition zones were observed for penicillin G, rifampicin, ciprofloxacin, or ceftazidime. Because MIC values and standardized breakpoint interpretations were not determined, these findings are reported solely as preliminary phenotypic inhibition-zone observations and were not used to classify the isolate as susceptible or resistant. Despite its probiotic-associated traits, the isolate did not exhibit measurable antimicrobial activity against the tested pathogens under the applied experimental conditions. No inhibition zones were observed in the well diffusion assay (Table 4 and Figure 2). Antimicrobial properties, one of the characteristics of probiotics, have also been investigated. Blhoney isolate was not effective against any of the bacteria tested.

Antibiotic susceptibility is another important criterion for evaluating the safety of potential probiotic strains. Phenotypic testing demonstrated measurable inhibition zones for several antibacterial agents under the experimental conditions. Because MIC values and standardized species-specific breakpoint interpretations were not determined, these findings are presented as preliminary inhibition-zone observations rather than definitive classifications of susceptibility or resistance. Antibiotic resistance among environmental bacterial isolates is commonly reported and may arise from intrinsic resistance mechanisms rather than acquired resistance genes [44]. Previous studies have also reported varying antibiotic susceptibility profiles among *Bacillus* strains isolated from food and environmental samples [28]. Furthermore, recent investigations have shown that *Bacillus* probiotic strains may exhibit resistance to one or more antibiotics while maintaining safety and probiotic functionality, although careful genomic evaluation is recommended to exclude transferable resistance genes [30]. In addition, several studies have demonstrated that *Bacillus* species used as probiotics frequently display strain-dependent antibiotic susceptibility patterns, highlighting the importance of evaluating antibiotic resistance during probiotic safety assessment [29,39].

Antibiotic susceptibility represents another critical criterion in the safety assessment of *Bacillus*-based probiotics. Previous investigations have shown that *B. licheniformis* isolates frequently exhibit heterogeneous susceptibility patterns, reflecting both intrinsic resistance mechanisms and strain-specific genetic characteristics [29,39]. The phenotypic susceptibility profile observed for Blhoney is therefore consistent with the variability previously reported among environmental *Bacillus* isolates. However, phenotypic susceptibility testing alone cannot distinguish intrinsic resistance from transferable antimicrobial resistance determinants. Accordingly, whole-genome sequencing and genomic characterization of resistance-associated genes are necessary to establish the safety profile of the isolate and should be considered an essential component of future investigations.

Although many probiotic bacteria exhibit antimicrobial activity against pathogenic microorganisms, the isolate investigated in this study did not demonstrate inhibitory activity against the tested pathogens. Similar findings have been reported in several studies where probiotic candidates did not produce detectable antimicrobial compounds under laboratory conditions [32]. In fact, antimicrobial activity among probiotic bacteria is often strain-specific, and not all isolates exhibit inhibitory effects against the tested microorganisms [40,41]. Some studies have shown that certain probiotic strains may not produce detectable inhibition zones against selected pathogens in standard agar diffusion assays despite possessing other probiotic properties such as gastrointestinal tolerance and metabolic activity [42]. Furthermore, it has been reported that the antimicrobial activity of probiotic bacteria can vary depending on the growth medium, incubation conditions, and the type of target microorganisms used in the assay [45].

The absence of antimicrobial activity may therefore be related to differences in experimental conditions, including nutrient availability, pH, or the metabolic state of the bacterial culture. It is also known that probiotic bacteria may exert beneficial effects through mechanisms other than direct pathogen inhibition, such as competitive exclusion, modulation of microbial communities, or stimulation of host immune responses rather than the production of antimicrobial compounds [40]. Therefore, the lack of detectable antimicrobial activity under the conditions tested should not be interpreted as evidence either for or against the preliminary probiotic-associated characteristics of Blhoney. The findings of the present study are limited to the specific phenotypic characteristics evaluated under the applied in vitro conditions and do not establish the overall probiotic potential of the isolate.

An additional limitation concerns the taxonomic resolution of the recovered isolates. Although the 20 isolates showed 16S rRNA gene sequence assignments consistent with *B. licheniformis*, 16S rRNA sequencing alone has limited discriminatory power for resolving closely related taxa and strain-level diversity within the *B. subtilis* group. Consequently, the present data cannot establish whether the recovered isolates represent clonal replicates or genetically distinct strains. Higher-resolution molecular approaches, particularly whole-genome sequencing or strain-typing methods such as ERIC-PCR or RAPD, are required to determine their genetic relatedness and confirm strain-level diversity.

Overall, the present study showed that bacterial isolation from honeys of different botanical origins yielded multiple isolates with 16S rRNA-based taxonomic assignments consistent with *B. licheniformis*. Detailed characterization of the Blhoney isolate demonstrated persistence under the tested simulated gastrointestinal stress conditions, supporting further investigation of its preliminary probiotic-associated characteristics. Although these findings should not be generalized to all recovered isolates without strain-level functional characterization, they demonstrate that honey can harbor resilient *B. licheniformis* strains and provide a basis for future comparative investigations of honey-derived *Bacillus* isolates. These findings support previous studies emphasizing the importance of bee products as reservoirs of beneficial microbes with potential applications in biotechnology, food science, and health-related fields [18,27,43].

This study demonstrated that honey could serve as a valuable ecological reservoir for microorganisms with potential probiotic characteristics. From the analyzed honey samples, a bacterial isolate identified as *Bacillus licheniformis* (Blhoney) was successfully obtained and characterized using molecular and microbiological approaches. The isolate exhibited persistence under the simulated gastrointestinal conditions tested, including exposure to acidic pH, bile salts, and digestive enzymes. These characteristics represent essential criteria for the selection of potential probiotic microorganisms [27,43,45].

Although the isolate did not show detectable antimicrobial activity against the tested pathogenic microorganisms under the experimental conditions, its persistence under the tested gastrointestinal stress conditions and its observed probiotic-associated characteristics support further investigation of its potential functional properties. The absence of antimicrobial activity may be associated with strain-specific characteristics or experimental conditions influencing metabolite production. The results of this study contribute to the growing body of evidence that bee-derived products, particularly honey, represent promising sources of beneficial microorganisms with potential applications in food biotechnology and functional product development. Moreover, the identification of *Bacillus licheniformis* from honey supports previous studies indicating that spore-forming *Bacillus* species may serve as stable and resilient probiotic candidates.

Although previous studies have isolated *Bacillus* species from honey and other bee-derived products, relatively few investigations have focused on the detailed in vitro characterization of honey-derived *B. licheniformis* isolates recovered from honeys of different botanical origins. In this context, the present study provides additional information regarding the gastrointestinal stress tolerance and phenotypic antibiotic inhibition-zone profile of the honey-derived Blhoney isolate. Nevertheless, the findings should be interpreted as an initial characterization rather than a comprehensive probiotic evaluation, and additional genomic, functional, and in vivo studies are required to fully establish the probiotic potential and safety of the Blhoney isolate.

The absence of detectable inhibition zones in the agar well diffusion assay should be interpreted cautiously. This method primarily detects diffusible extracellular antimicrobial compounds and may underestimate antagonistic mechanisms requiring direct cell-to-cell interactions or metabolites produced only under specific environmental conditions. In *Bacillus licheniformis*, the synthesis of antimicrobial compounds, including lipopeptides such as lichenysin, as well as organic acids and other secondary metabolites, is known to be highly strain-dependent and strongly influenced by nutrient availability, growth phase, oxygen availability, and cultivation conditions. Because neither metabolite production nor the presence of biosynthetic gene clusters responsible for antimicrobial compound synthesis was investigated in the present study, the lack of inhibition zones cannot be interpreted as evidence that the isolate lacks antimicrobial potential. Future studies employing co-culture assays, metabolomic profiling, and whole-genome sequencing to investigate biosynthetic gene clusters, including the lichenysin operon, will provide a more comprehensive assessment of the antimicrobial capacity of the Blhoney isolate.

Although *Bacillus* species have previously been isolated from honey and other bee-derived products, relatively few studies have focused on the detailed in vitro characterization of honey-derived *B. licheniformis* isolates originating from botanically distinct honey samples. The present study therefore contributes additional information regarding the gastrointestinal stress tolerance and phenotypic antibiotic inhibition-zone profile of a representative honey-derived *B. licheniformis* isolate. Nevertheless, the findings should be regarded as preliminary, and further genomic and functional investigations are required to establish its probiotic safety and efficacy.

The present findings should be interpreted within the scope of an initial in vitro assessment of the preliminary probiotic-associated characteristics of Blhoney. Although gastrointestinal stress tolerance, phenotypic antibiotic inhibition-zone profile, and antimicrobial activity were evaluated, genomic safety characterization was not performed. The antibiotic susceptibility profile presented in this study is based on phenotypic susceptibility testing and should therefore be regarded as a preliminary assessment. Future investigations should incorporate minimum inhibitory concentration (MIC) determinations in accordance with internationally accepted standards, together with whole-genome sequencing to identify antimicrobial resistance determinants and evaluate their potential transferability.

In particular, the presence of virulence-associated determinants and transferable antimicrobial resistance genes was not investigated. Therefore, the current findings should not be considered a comprehensive assessment of the probiotic safety of the strain. Further studies incorporating whole-genome sequencing and genomic screening for virulence and antimicrobial resistance determinants, together with additional functional and in vivo assessments, are required before Blhoney can be considered for potential probiotic applications.

It should be emphasized that the potential application of Blhoney as a probiotic is not associated with the direct consumption of honey. Honey naturally contains only low numbers of viable microorganisms, and these populations may further decline during processing and storage. Instead, honey should be regarded as a natural ecological reservoir for the isolation of promising bacterial strains. Following isolation, candidate strains such as Blhoney could be propagated under controlled laboratory and industrial fermentation conditions, subjected to comprehensive safety evaluations, and subsequently developed into probiotic formulations if their safety and efficacy are confirmed through further genomic and in vivo studies.

## 3. Materials and Methods

### 3.1. Honey Samples and Test Bacteria

In this study, cultivable bacterial isolates were recovered from four different honey samples harvested during the 2025 production season. The names, sample codes, and selected characteristics of the analyzed honey samples are presented in Table 1. Melissopalynological analysis based on microscopic pollen examination was performed to determine the botanical origin of the honey samples [46]. Determination of botanical origin was achieved via light microscopic evaluation of pollen morphology, whereas quantitative analysis of pollen content was conducted using Lycopodium spores as a reference indicator. Based on pollen composition, the samples were grouped into four categories: dominant pollen (>45%), indicating monofloral honey; secondary pollen (16–45%); minor pollen (3–15%); and trace levels (<3%) [25].

### 3.2. Determination of Physicochemical Properties

The Moisture and electrical conductivity (EC) of the honey samples were measured following the harmonized honey methods [47]. The specific optical rotation of the honey samples was measured using a polarimeter (Kruss Optical Activity, Hamburg, Germany) after clarification with Carrez reagents I and II [48]. Turbidity was determined using a turbidimeter (WTW Turb 550, WTW, Munich, Germany). The instrument was calibrated with formazan standard suspensions (1000, 10, and 0.002 NTU) covering the measurement range, and turbidity values were recorded according to Standard Methods (SM 2130B, 2017).

Proline content in the honey samples was determined by HPLC–UV following a derivatization reaction with ninhydrin, which forms a colored complex with amino acids [49]. The results were expressed as mg/kg. Total phenolic content was determined using the Folin–Ciocalteu method. Phenolic compounds in the sample reduce the Folin reagent, forming a purple–violet complex with maximum absorbance at 765 nm [50]. Quantification was performed using a gallic acid calibration curve, and the results were expressed as mg GAE/100 g honey.

### 3.3. Test Bacteria

A diverse panel of reference microorganisms, including Gram-positive bacteria, Gram-negative bacteria, and a yeast, was employed to examine the antimicrobial activity of the isolate. The test microorganisms consisted of *Escherichia coli* (ATCC 25922), *Enterobacter cloacae* (ATCC 13047), *Salmonella typhimurium* (ATCC 14028), *Serratia marcescens*, *Proteus vulgaris* (ATCC 13315), *Klebsiella pneumoniae* (ATCC 13883), *Staphylococcus epidermidis* (ATCC 12228), *Enterococcus faecalis* (ATCC 29212), *Bacillus thuringiensis*, *Staphylococcus aureus* (ATCC 25923), and *Candida albicans* (ATCC 10231).

All strains were obtained from the culture collection of the host institution and maintained under appropriate laboratory conditions prior to use. Prior to use, the microorganisms were reactivated and maintained under appropriate laboratory conditions to ensure experimental consistency.

### 3.4. Isolation and Preliminary Identification of Bacteria from Honey Samples

For the isolation of bacterial populations, 1 g of each honey sample was aseptically diluted in 10 mL of sterile de Man, Rogosa, and Sharpe (MRS) broth and homogenized using a vortex mixer (LVOM-A12, Surrey, UK). The suspensions were incubated at 30 °C for 3–7 days under static conditions to facilitate the enrichment of honey-associated microorganisms. Following the enrichment stage, aliquots were streaked onto MRS agar supplemented with 0.8% (*w*/*v*) CaCO_3_ and 2% (*w*/*v*) fructose. The incorporation of calcium carbonate enabled the visualization of acid-producing colonies through the formation of transparent halos caused by CaCO_3_ dissolution. Incubation was carried out at 30 °C for 72–96 h in an oxygen-free environment provided by an anaerobic system. Although MRS medium is widely used for the enrichment of lactic acid bacteria, it may also support the growth of other acid-producing bacteria. Therefore, the use of MRS medium in the present study was intended to enrich acid-producing microorganisms rather than to selectively isolate LAB. The taxonomic identity of the recovered isolate was subsequently confirmed by 16S rRNA gene sequencing.

Colonies exhibiting distinct clear zones were selected as potential acid-producing bacteria and sub-cultured repeatedly to obtain pure isolates. Following purification, a total of 20 bacterial isolates were obtained: 4 from H1, 5 from H2, 6 from H3, and 5 from H4. One isolate, designated Blhoney, originating from H1, was selected for subsequent molecular and functional characterization. Purified cultures were maintained on MRS agar at 4 °C for short-term use and preserved in 20% glycerol at −80 °C for long-term storage [51]. Preliminary identification was performed using Gram staining and catalase testing to determine phenotypic characteristics.

### 3.5. Molecular Identification of Bacterial Isolate

Molecular identification of the recovered bacterial isolates was performed based on 16S rRNA gene sequencing. Genomic DNA was extracted according to previously established protocols [52], and the 16S rRNA gene was amplified using universal bacterial primers (F-5′-ATTCTAGAGTTTGATCATGGCTCA-3′ and R-5′-ATGGTACCGTGTGACGGGCGGTGTGTA-3′) [33]. Sequence comparison against the NCBI GenBank database using BLAST analysis showed that the 16S rRNA gene sequences of the recovered isolates had their closest taxonomic assignments to *Bacillus licheniformis*. Because 16S rRNA gene sequencing does not provide sufficient resolution to establish strain-level diversity or clonality within the *Bacillus subtilis*/*licheniformis* group, no inference regarding the genetic distinctness of the 20 isolates was made. One isolate, designated Blhoney, was selected for subsequent detailed molecular and functional characterization. The approximately 1500-bp 16S rRNA gene sequence of Blhoney was used for further taxonomic analysis and deposited in GenBank under accession number PZ120893.1.

Amplified products were visualized on 1% agarose gel electrophoresis and subsequently sequenced. The obtained sequences were compared against the NCBI GenBank database using BLAST (version BLAST+ 2.17.0 ) analysis [53]. Approximately 1500 bp of the 16S rRNA gene sequence was used for similarity assessment and taxonomic identification [52].

### 3.6. Scanning Electron Microscopy (SEM) of Bacterial Sample

The morphological characteristics of the bacterial isolate (Blhoney) were examined using scanning electron microscopy (SEM) (Carl Zeiss, EVO LS 10, Oberkochen, Germany). For this purpose, the isolate was first cultivated on nutrient agar at 30 °C for 24 h. Bacterial cells were carefully collected from the agar surface using sterile inoculating loops and suspended in deionized water. The suspension was homogenized and subjected to centrifugation at 7500 rpm for 5 min. The washing procedure was repeated multiple times to remove residual media components. Following purification, the bacterial suspension was diluted appropriately, and a small volume (5 μL) was placed onto a SEM stub and allowed to air-dry. The samples prepared were analyzed using a scanning electron microscope (Carl Zeiss, EVO LS 10, Oberkochen, Germany) under an accelerating voltage ranging between 5 and 10 kV.

### 3.7. Probiotic-Associated Characteristics of the Blhoney Isolate

The probiotic potential of the isolate was evaluated by assessing its tolerance to simulated gastrointestinal conditions [54]. Actively cultured bacterial cells were collected and purified using phosphate-buffered saline (PBS), followed by exposure to a range of stress conditions, including acidic environments (pH 1–3), bile salts (0.3–1%), pepsin, and pancreatin.

Simulated gastric fluid consisted of phosphate-buffered saline adjusted to pH 2.0 and supplemented with pepsin (3 mg/mL). Simulated intestinal fluid consisted of phosphate-buffered saline adjusted to pH 8.0 and supplemented with pancreatin (1 mg/mL). An inoculum of approximately 1 × 10^8^ CFU/mL was used for each assay.

The treated samples were incubated at 37 °C for varying durations, and bacterial survival was determined both spectrophotometrically (OD_600_) and by viable cell counts (CFU/mL). These assays were performed to evaluate the ability of the isolate to withstand conditions mimicking the gastrointestinal environment [55].

The survival rate (%) was calculated by comparing the viable cell counts (CFU/mL) obtained after exposure to each simulated gastrointestinal condition with those of the corresponding untreated control cultures according to the following equation:

Survival (%) = (CFU after treatment/CFU before treatment) × 100

Optical density (OD_600_) measurements were used to support the viable count results.

### 3.8. Phenotypic Antibiotic Inhibition-Zone Assay

The phenotypic antibiotic inhibition-zone profile of the isolate was evaluated using an agar diffusion assay under the experimental conditions described below [56]. Following standardization to approximately 10^8^ CFU/mL (0.5 McFarland), the bacterial suspension was evenly inoculated onto agar plates, and antibiotic discs were then aseptically placed on the surface. The plates were incubated at 37 °C for 16–18 h, after which the diameters of inhibition zones surrounding each disc were measured. Commercial antibiotic discs (Oxoid, Basingstoke, UK) containing ampicillin (10 μg/disc), chloramphenicol (30 μg/disc), erythromycin (15 μg/disc), gentamicin (10 μg/disc), kanamycin (30 μg/disc), spectinomycin (100 μg/disc), streptomycin (10 μg/disc), tetracycline (30 μg/disc), penicillin G (10 units/disc), rifampicin (5 μg/disc), ciprofloxacin (5 μg/disc) and ceftazidime (30 μg/disc) were used according to the manufacturer’s instructions. Inhibition-zone diameters were measured in three independent replicates for each antibacterial agent and are reported descriptively as mean ± SD. No inferential statistical comparisons were performed among different antibacterial agents because of differences in disc potency, diffusion characteristics, and mechanisms of action.

### 3.9. Analysis of Antimicrobial Potential

A well diffusion approach was employed to examine antimicrobial activity, utilizing cell-free supernatants obtained after centrifugation and sterile filtration (0.22 μm). Indicator microorganisms were cultured and spread onto agar plates to form a uniform lawn. Wells were created aseptically, and aliquots of the supernatant were introduced into each well. Following incubation, inhibition zones were measured to assess antimicrobial potential [57].

## 4. Conclusions

This study demonstrated that honey could serve as an ecological reservoir for resilient bacterial isolates with potential functional significance. A total of 20 bacterial isolates were recovered from four honey samples representing different botanical origins. Based on 16S rRNA gene sequence analysis, the recovered isolates showed taxonomic assignments consistent with *Bacillus* licheniformis. However, because 16S rRNA sequencing provides limited resolution for strain-level discrimination within the *Bacillus subtilis* species group, the clonality and genetic diversity of these isolates could not be established. One isolate, designated Blhoney, was selected for subsequent functional characterization.

Blhoney exhibited strong tolerance to simulated gastrointestinal conditions, including acidic pH, bile salts, and digestive enzymes, supporting further investigation of its preliminary probiotic-associated characteristics. Although no detectable antimicrobial activity against the tested microorganisms was observed under experimental conditions, this finding does not exclude other probiotic-associated mechanisms or functional properties. Importantly, the functional findings obtained by Blhoney should be interpreted as strain-specific and cannot be directly generalized to the remaining *B. licheniformis* isolates without further comparative characterization.

Overall, the findings demonstrate the recovery of bacterial isolates with 16S rRNA-based taxonomic assignments consistent with *B. licheniformis* from honey samples of different botanical origins and describe preliminary probiotic-associated characteristics of the Blhoney isolate under the tested in vitro conditions. These findings should not be interpreted as demonstrating the probiotic safety or efficacy of Blhoney. Further studies incorporating whole-genome sequencing, standardized MIC-based antimicrobial susceptibility testing, antimicrobial-resistance and virulence/toxigenicity gene analysis, differentiation of vegetative cells from endospores, and appropriate in vivo models are required before its safety and potential probiotic application can be established.

## Figures and Tables

**Table 1:** Honey samples, codes, and botanical origins of the analyzed honey samples.

Parameters	H1	H2	H3	H4
Region/Türkiye	Çanakkale	Trabzon/Şalpazarı	Balıkesir	Rize/İyidere
Honey Origin	Honeydew	Blossom	Blossom	Honeydew
Dominant pollen > 45%	-	*Rhododendron* ssp. 70%	Dominant pollen > 45%	-
Secondary pollen > 20–45%	*Helianthus* spp., Rhamnaceae, Rosaceae, *Hedera* spp.	*Chestnut sativa* *Myosotis* spp. *Cephalaria* spp. *Hedysarum* spp. Poaceae Rosaceae	*Helianthus* spp. *Pinus* spp. Amaryllidaceae *Trifolium* spp. *Calluna* spp.	-
Minor pollen < 20%	*Quercus* spp., Ericaceae	*Carex* spp. *Plantago* spp.	*Salix* spp. Brassicaceae Ericaceae	Ericaceae, *Calluna vulgaris*, *Rhododendron* spp. *Quercus* spp.

**Table 2:** Physicochemical properties of the honey samples.

Parameters	H1	H2	H3	H4
Moisture (g/100 g)	18.00 ± 0.04 ^a^	18.00 ± 0.05 ^a^	17.50 ± 0.05 ^a^	18.00 ± 0.05 ^a^
Electrical conductivity EC (mS/cm)	0.95 ± 0.02 ^a^	0.42 ± 002 ^b^	0.34 ± 0.02 ^c^	1.24 ± 0.20 ^d^
Specific optic rotations [α]	0.85 ± 0.02 ^a^	−1.80 ± 0.05 ^b^	−1.56 ± 0.02 ^c^	+2.56 ± 0.20 ^d^
Proline (mg/kg)	746.20 ± 12.25 ^a^	564.22 ± 5.44 ^b^	480.25 ± 6.71 ^c^	388.25 ± 4.68 ^d^
Total Phenolic Contents (mg GAE/100 g)	72.24 ± 4.20 ^a^	31.64 ± 1.44 ^b^	27.52 ± 3.22 ^c^	35.44 ± 3.05 ^d^

All values presented in the table are expressed as the mean of three replicates. Different letters (a, b, c, d) indicate statistically significant differences among the honey samples at *p* < 0.05, as determined by One-Way ANOVA.

**Table 3:** Percentage survival of *Bacillus licheniformis* (Blhoney) under in vitro gastrointestinal stress factors.

Stress Condition	1 h Survival (%)	3 h Survival (%)	24 h Survival (%)
pH 1.0	98.37 ± 0.31	98.23 ± 0.25	97.53 ± 0.31
pH 2.0	97.30 ± 0.44	97.57 ± 0.31	97.30 ± 0.30
pH 3.0	99.30 ± 0.42	98.90 ± 0.71	99.05 ± 0.49
Bile salts (0.3%)	98.53 ± 0.45	98.30 ± 0.36	98.17 ± 0.15
Bile salts (0.5%)	98.90 ± 0.46	98.53 ± 0.51	98.37 ± 0.55
Bile salts (1.0%)	98.57 ± 0.60	98.30 ± 0.85	98.10 ± 1.00
Pepsin	99.07 ± 0.31	98.67 ± 0.46	98.47 ± 0.55
Pancreatin	99.20 ± 0.28	98.80 ± 0.57	98.50 ± 0.71

**Table 4:** Phenotypic inhibition-zone measurements of the Blhoney isolate against tested antibacterial agents. (zone diameter, mm).

Isolate ID	Antibiotic Agent (µg/mL)	Inhibition Zone (mm)
Blhoney	Erythromycin	26.41 ± 1.10
Blhoney	Kanamycin	24.02 ± 1.16
Blhoney	Chloramphenicol	60.30 ± 0.55
Blhoney	Ampicillin	19.00 ± 0.57
Blhoney	Tetracycline	46.30 ± 1.52
Blhoney	Gentamicin	20.00 ± 1.00
Blhoney	Streptomycin	20.50 ± 0.57
Blhoney	Spectinomycin	50.00 ± 1.52

Values are presented as mean ± SD from three independent replicate measurements for each antibacterial agent. Only antibacterial agents producing measurable inhibition zones are presented. Because the tested agents differ in disc potency, diffusion characteristics, physicochemical properties, and mechanisms of action, inhibition-zone diameters were not statistically compared across antibacterial agents. MIC determination and standardized breakpoint interpretation were not performed; therefore, the data are reported solely as preliminary phenotypic inhibition-zone observations and were not used to classify the isolate as susceptible or resistant. (Only antibiotics with measurable inhibition zones are shown. Antibiotics producing no inhibition zone are not presented in this table. The inhibition zone data presented represent phenotypic observations only. No MIC determination or breakpoint interpretation according to EUCAST or CLSI guidelines was performed).

## Data Availability

The datasets were generated or analyzed during the current study. The raw data supporting the conclusions of this article will be made available by the authors on request. The 16S rRNA gene sequence of the Blhoney isolate has been deposited in GenBank under accession number PZ120893.1.

## Abbreviations

The following abbreviations are used in this manuscript:

HPLC-UV	High-Performance Liquid Chromatography with Ultraviolet Detection
GAE	Gallic acid equivalent
LAB	Lactic acid bacteria
EC	Electrical conductivity
CFU	Colony-Forming Unit
SEM	Scanning electron microscopy
TPC	Total Phenolic Content
PCR	Polymerase Chain Reaction

## References

[1] Bose D., Padmavati M. (2024). Honey Authentication: A Review of the Issues and Challenges Associated with Honey Adulteration. Food Biosci..

[2] Alvarez-Suarez J.M., Tulipani S., Romandini S., Bertoli E., Battino M. (2010). Contribution of Honey in Nutrition and Human Health: A Review. Mediterr. J. Nutr. Metab..

[3] Snowdon J.A., Cliver D.O. (1996). Microorganisms in Honey. Int. J. Food Microbiol..

[4] Ogwu M.C., Izah S.C. (2025). Honey as a Natural Antimicrobial. Antibiotics.

[5] Nolan V.C., Harrison J., Cox J.A. (2019). Dissecting the antimicrobial composition of honey. Antibiotics.

[6] Gilliam M. (1997). Identification and Roles of Non-Pathogenic Microflora Associated with Honeybees. FEMS Microbiol. Lett..

[7] Olofsson T.C., Vásquez A. (2008). Detection and Identification of a Novel Lactic Acid Bacterial Flora within the Honey Stomach of the Honeybee *Apis mellifera*. Curr. Microbiol..

[8] Vásquez A., Forsgren E., Fries I., Paxton R.J., Flaberg E., Szekely L., Olofsson T.C. (2012). Symbionts as Major Modulators of Insect Health: Lactic Acid Bacteria and Honeybees. PLoS ONE.

[9] FAO/WHO Expert Committee on Food Additives (2002). Evaluation of Certain Food Additives and Contaminants: Fifty-Seventh Report of the Joint FAO/WHO Expert Committee on Food Additives.

[10] Anderson K.E., Sheehan T.H., Eckholm B.J., Mott B.M., DeGrandi-Hoffman G. (2011). An Emerging Paradigm of Colony Health: Microbial Balance of the Honey Bee and Hive (*Apis mellifera*). Insectes Soc..

[11] Bonilla-Rosso G., Engel P. (2018). Functional Roles and Metabolic Niches in the Honeybee Gut Microbiota. Curr. Opin. Microbiol..

[12] Alparslan B., Demirci A., Birinci C., Tekintaş Y., Kolaylı S. (2025). Biochemical Characterization and Functional Properties of Gezo Honeydew Honey Derived from *Salix* spp. in Eastern Anatolia. J. Food Meas. Charact..

[13] Sahin H., Ozkok A., Tanugur Samanci A.E., Onder E.Y., Kolayli S. (2022). Identification of the main phenolic markers in Turkish pine honeys and their biological functions. Chem. Biodivers..

[14] Zengin K., Usta M., Okuyan S., Solmaz S., Nalcacioglu R., Demirbağ Z. (2025). Comparative Evaluation of the Antimicrobial Activities of Monofloral Honeys from Diverse Botanical Origins. J. Apither. Nat..

[15] Douglas A.E. (2006). Phloem-Sap Feeding by Animals: Problems and Solutions. J. Exp. Bot..

[16] Álvarez-Pérez S., Lievens B., de Vega C. (2024). Floral Nectar and Honeydew Microbial Diversity and Their Role in Biocontrol of Insect Pests and Pollination. Curr. Opin. Insect Sci..

[17] Luca L., Pauliuc D., Ursachi F., Oroian M. (2025). Physicochemical Parameters, Microbiological Quality, and Antibacterial Activity of Honey from the Bucovina Region of Romania. Sci. Rep..

[18] Grabek-Lejko D., Worek M. (2024). Honeydew Honey as a Reservoir of Bacteria with Antibacterial and Probiotic Properties. Antibiotics.

[19] Pląder M., Sękul J., Kot A.M., Pobiega K. (2025). From Hive to Laboratory—Biotechnological Potential of Microorganisms from Honey. World J. Microbiol. Biotechnol..

[20] Kemal M., Esertaş Üzü E., Kanbur E.D., Kara Y., Özçelik A.E., Can Z., Kolaylı S. (2023). Characterization of the Black Cumin (*Nigella sativa* L.) Honey from Türkiye. Food Biosci..

[21] Dursun I., Kanbur E.D., Kara Y., Kolaylı S. (2025). Determination of Grayanotoxin-III Amount in the Rhododendron Honey and Flowers Samples Using UHPLC-Orbitrap^®^-HRMS. Toxicon.

[22] Çakır H.E., Malkoç M.E., Şahin H., Kara Y., Kolaylı S. (2023). An Investigation of the Antihypertensive Effect of Mad Honey and *Rhododendron luteum* Sweet Extract Induced by N-ω-Nitro-L-Arginine Methyl Ester (L-NAME) in Rats. Indian J. Tradit. Knowl..

[23] Ucurum O., Tosunoglu H., Takma Ç., Birlik P.M., Berber M., Kolaylı S. (2024). Distinctive Properties of the Pine, Oak, Chestnut and Multifloral Blossom and Honeydew Honeys. Eur. Food Res. Technol..

[24] Birinci C., Kemal M., Kanbur E.D., Kara Y., Özçelik A.E., Kolaylı S. (2026). Physicochemical, Biochemical, and Chemometric Characterization of Oak Honey (*Quercus* spp.) from Kırklareli, Türkiye. Chem. Biodivers..

[25] Kolaylı S., Kazaz G., Özkök A., Keskin M., Kara Y., Demir Kanbur E., Ertürk Ö. (2023). The Phenolic Composition, Aroma Compounds, Physicochemical and Antimicrobial Properties of *Nigella sativa* L. (Black Cumin) Honey. Eur. Food Res. Technol..

[26] Ptaszyńska A.A., Latoch P., Hurd P.J., Polaszek A., Michalska-Madej J., Grochowalski Ł., Strapagiel D., Gnat S., Załuski D., Gancarz M. (2021). Amplicon Sequencing of Variable 16S rRNA from Bacteria and ITS2 Regions from Fungi and Plants Reveals Honeybee Susceptibility to Diseases Results from Their Forage Availability under Anthropogenic Landscapes. Pathogens.

[27] Kwong W.K., Moran N.A. (2016). Gut Microbial Communities of Social Bees. Nat. Rev. Microbiol..

[28] Elshaghabee F.M., Rokana N., Gulhane R.D., Sharma C., Panwar H. (2017). Bacillus as Potential Probiotics: Status, Concerns, and Future Perspectives. Front. Microbiol..

[29] Lee N.K., Kim W.S., Paik H.D. (2019). Bacillus Strains as Human Probiotics: Characterization, Safety, Microbiome, and Probiotic Carrier. Food Sci. Biotechnol..

[30] Ramirez-Olea H., Reyes-Ballesteros B., Chavez-Santoscoy R.A. (2022). Potential Application of the Probiotic *Bacillus licheniformis* as an Adjuvant in the Treatment of Diseases in Humans and Animals: A Systematic Review. Front. Microbiol..

[31] Li J., Tian C., Feng S., Cheng W., Tao S., Li C., Wei H. (2024). Modulation of Gut Microbial Community and Metabolism by *Bacillus licheniformis* HD173 Promotes the Growth of Nursery Piglets. Nutrients.

[32] Feizabadi F., Sharifan A., Tajabadi N. (2021). Isolation and Identification of Lactic Acid Bacteria from Stored *Apis mellifera* Honey. J. Apic. Res..

[33] Usta M., Zengin K., Okuyan S., Solmaz S., Nalçacıoğlu R., Demirbağ Z. (2025). Isolation and Probiotic Evaluation of *Apilactobacillus kunkeei* and *Bombella* sp. from *Apis mellifera anatoliaca* and *Bombus terrestris*. Int. Microbiol..

[34] Araya M., Morelli L., Reid G., Sanders M.E., Stanton C., Pineiro M., Ben Embarek P. (2002). Guidelines for the Evaluation of Probiotics in Food.

[35] Yu X., Dai Z., Cao G., Cui Z., Zhang R., Xu Y., Yang C. (2023). Protective Effects of *Bacillus licheniformis* on Growth Performance, Gut Barrier Functions, Immunity and Serum Metabolome in Lipopolysaccharide-Challenged Weaned Piglets. Front. Immunol..

[36] Cutting S.M. (2011). Bacillus Probiotics. Food Microbiol..

[37] Iorizzo M., Letizia F., Ganassi S., Testa B., Petrarca S., Albanese G., De Cristofaro A. (2022). Functional Properties and Antimicrobial Activity from Lactic Acid Bacteria as Resources to Improve the Health and Welfare of Honeybees. Insects.

[38] Suyabatmaz Ş., Karaoğlu Ş.A., Bozdeveci A., Akpınar R. (2023). Honeybee-Associated Lactic Acid Bacteria and Their Probiotic Potential for Human Use. World J. Microbiol. Biotechnol..

[39] Jin X., Zhang L., Cao Y., Dai Z., Ge X., Cai R., Hu Z. (2025). Antibiotic Resistance Characterization, Virulence Factors and Molecular Characteristics of *Bacillus* Species Isolated from Probiotic Preparations in China. J. Glob. Antimicrob. Resist..

[40] Fijan S., Šulc D., Steyer A. (2018). Study of the In Vitro Antagonistic Activity of Various Single-Strain and Multi-Strain Probiotics against *Escherichia coli*. Int. J. Environ. Res. Public Health.

[41] Prabhurajeshwar C., Chandrakanth K. (2019). Evaluation of Antimicrobial Properties and Their Substances against Pathogenic Bacteria In Vitro by Probiotic *Lactobacilli* Strains Isolated from Commercial Yoghurt. Clin. Nutr. Exp..

[42] Barzegar H., Alizadeh Behbahani B., Falah F. (2021). Safety, Probiotic Properties, Antimicrobial Activity, and Technological Performance of *Lactobacillus* Strains Isolated from Iranian Raw Milk Cheeses. Food Sci. Nutr..

[43] Payne J., Bellmer D., Jadeja R., Muriana P. (2024). The Potential of *Bacillus* Species as Probiotics in the Food Industry: A Review. Foods.

[44] Rychen G., Aquilina G., Azimonti G., Bampidis V., de Lourdes Bastos M., Bories G., Chesson A., Cocconcelli P.S., Flachowsky G., EFSA Panel on Additives and Products or Substances Used in Animal Feed (FEEDAP) (2018). Guidance on the Characterization of Microorganisms Used as Feed Additives or as Production Organisms. EFSA J..

[45] Vuran Ö., Karagözlü N., Akpınar A. (2021). The Antimicrobial Effects of Probiotic and Traditional Yoghurts Produced Using Commercial Starter Cultures on Some Foodborne Pathogens. J. Agric. Fac. Ege Univ..

[46] Louveaux J., Maurizio A., Vorwohl G. (1978). Methods of Melissopalynology. Bee World.

[47] Bogdanov S., Martin P., Lüllmann C. (1997). Harmonised Methods of the European Honey Commission. Apidologie.

[48] Piazza M.G., Accorti M., Persano Oddo L. (1991). Electrical Conductivity, Ash, Colour and Specific Rotatory Power in Italian Unifloral Honeys. Apicoltura.

[49] Ough C.S. (1969). Rapid determination of proline in grapes and wines. J. Food Sci..

[50] Singleton V.L., Orthofer R., Lamuela-Raventós R.M., Packer L. (1999). Analysis of Total Phenols and Other Oxidation Substrates and Antioxidants by Means of Folin–Ciocalteu Reagent. Methods in Enzymology.

[51] O’Toole P.W., Marchesi J.R., Hill C. (2017). Next-Generation Probiotics: The Spectrum from Probiotics to Live Biotherapeutics. Nat. Microbiol..

[52] Sambrook J., Fritsch E.F., Maniatis T. (1989). Molecular Cloning: A Laboratory Manual.

[53] Altschul S.F., Gish W., Miller W., Myers E.W., Lipman D.J. (1990). Basic Local Alignment Search Tool. J. Mol. Biol..

[54] Jamaly N., Benjouad A., Bouksaim M. (2011). Probiotic Potential of *Lactobacillus* Strains Isolated from Known Popular Traditional Moroccan Dairy Products. Br. Microbiol. Res. J..

[55] Maragkoudakis P.A., Zoumpopoulou G., Miaris C., Kalantzopoulos G., Pot B., Tsakalidou E. (2006). Probiotic Potential of *Lactobacillus* Strains Isolated from Dairy Products. Int. Dairy J..

[56] European Committee on Antimicrobial Susceptibility Testing (EUCAST) (2023). Breakpoint Tables for Interpretation of MICs and Zone Diameters.

[57] Sharma S., Gupta P., Kumar A., Ray J., Aggarwal B.K., Goyal P., Sharma A. (2014). In Vitro Evaluation of Roots, Seeds and Leaves of *Sesamum indicum* L. for Their Potential Antibacterial and Antioxidant Properties. Afr. J. Biotechnol..

